# Silencing the *SpMPK1*, *SpMPK2*, and *SpMPK3* Genes in Tomato Reduces Abscisic Acid—Mediated Drought Tolerance

**DOI:** 10.3390/ijms141121983

**Published:** 2013-11-06

**Authors:** Cui Li, Jian-Min Yan, Yun-Zhou Li, Zhen-Cai Zhang, Qiao-Li Wang, Yan Liang

**Affiliations:** 1 State Key Laboratory of Crop Stress Biology in Arid Region, Northwest A&F University, Yangling 712100, Shaanxi, China; E-Mails: lovelicui@nwsuaf.edu.cn (C.L.); yjmqhd0201@163.com (J.-M.Y.); liyunzhou2007@126.com (Y.-Z.L.); zhangzhencai0417@nwsuaf.edu.cn (Z.-C.Z.); wangql0704@163.com (Q.-L.W.); 2 College of Horticulture, Northwest A&F University, Yangling 712100, Shaanxi, China

**Keywords:** *Solanum pimpinellifolium*, protein kinase, stomata, drought tolerance, virus-induced gene silencing (VIGS)

## Abstract

Drought is a major threat to agriculture production worldwide. Mitogen-activated protein kinases (MAPKs) play a pivotal role in sensing and converting stress signals into appropriate responses so that plants can adapt and survive. To examine the function of MAPKs in the drought tolerance of tomato plants, we silenced the *SpMPK1*, *SpMPK2*, and *SpMPK3* genes in wild-type plants using the virus-induced gene silencing (VIGS) method. The results indicate that silencing the individual genes or co-silencing *SpMPK1*, *SpMPK2*, and *SpMPK3* reduced the drought tolerance of tomato plants by varying degrees. Co-silencing *SpMPK1* and *SpMPK2* impaired abscisic acid (ABA)-induced and hydrogen peroxide (H_2_O_2_)-induced stomatal closure and enhanced ABA-induced H_2_O_2_ production. Similar results were observed when silencing *SpMPK3* alone, but not when *SpMPK1* and *SpMPK2* were individually silenced. These data suggest that the functions of *SpMPK1* and *SpMPK2* are redundant, and they overlap with that of *SpMPK3* in drought stress signaling pathways. In addition, we found that *SpMPK3* may regulate H_2_O_2_ levels by mediating the expression of *CAT1*. Hence, *SpMPK1*, *SpMPK2*, and *SpMPK3* may play crucial roles in enhancing tomato plants’ drought tolerance by influencing stomatal activity and H_2_O_2_ production via the ABA-H_2_O_2_ pathway.

## 1. Introduction

The tomato plant, *Solanum lycopersicum*, is extensively cultivated and consumed around the world and therefore constitutes a major agricultural industry. Adequate water supply is a major concern for this industry because drought conditions impair the quality and yield of tomatoes by hindering the growth and development of the seedlings. Many signaling pathways are involved in mediating plants’ responses to drought stress. Mitogen-activated protein kinase (MAPK) cascade, the foundation of signal transduction networks, plays crucial roles in the ability of plants to tolerate and adapt to stresses by regulating stress signal transduction and the expression of relevant genes.

The MAPK signaling pathway is a three-tiered phosphorelay cascade consisting of MAPKs (MPKs), which are activated by MAPK kinases (MPKK or MKKs), which in turn are activated by MAPKK kinases (MAPKKKs). As the last component of the cascade to be activated, MAPKs can phosphorylate specific serine/threonine residues on the target protein, thereby regulating a variety of cellular activities [1]. An increasing body of evidence suggests, that in plants, MAPK cascades are involved in numerous developmental processes as well as signaling networks associated with stress responses, including tolerance to certain stressors [2–6]. Lampard and Wang reported that the MAPK kinases *AtMKK4*, *AtMKK5*, *AtMKK7*, and *AtMKK9* regulate stomatal development by phosphorylating *AtMPK3*, *AtMPK6*, and other unknown MAPK genes [7,8]. Because stomata are channels for gas exchange and water evaporation, their movement is crucial for plants to adapt to prevailing environmental conditions. Thus, the ability of MAPK cascades to regulate stomatal development implies that they may be involved in shaping plants’ stress tolerance. Several studies have found that MAPK cascades affect plants’ innate immunity to biotic stresses, such as bacteria, insects, and aphids [9–11]. Additional studies suggest that MAPK cascades are involved in processes regulated by the plant hormone, abscisic acid (ABA), when plants are exposed to various abiotic stressors[12].

ABA is a universal hormone in plants and plays a major role in plant growth and development, including embryo maturation, seed dormancy and germination, seedling establishment, and root branching. As a stress hormone, ABA regulates how plants respond to a variety of stresses by transmitting stress signals to the appropriate cells. These cells then activate the expression of stress-responsive genes and elicit other physiological responses, such as hydrogen peroxide (H_2_O_2_) production and stomatal movement induction. In *Arabidopsis* species, *AtMPKK1*, *AtMPKK3*, and *AtMPKK9* as well as *AtMPK1*, *AtMPK2*, *AtMPK3*, and *AtMPK6* play positive roles in ABA signaling during seed germination, and *MdMKK1* and *MdMPK1* function similarly in apples [13–19]. In addition, MAPKs have been implicated in ABA-induced antioxidant defenses. The *AtMEKK1*-*AtMKK1/AtMKK2*-*AtMPK4* cascade and the *AtMKK1*-*AtMPK6* signaling pathway play important roles in mitigating the effects of reactive oxygen species (ROS) [15,17,20–23]. In addition, previous studies have shown that the ABA-induced activity of guard cells, which are responsible for allowing or preventing gas exchange to occur through stomata, is mediated by MAPK cascades when abiotic stresses are present. In *Arabidopsis*, it was found that inhibiting *AtMPK3* caused stomata to partially lose sensitivity to ABA [24,25]. Similar behavior was found in *atmpk9/atmpk12* double mutants, which became insensitive to ABA-induced stomatal closure and ABA-inhibited stomatal opening [26]. Therefore, crosstalk exists between ABA signaling and MAPK cascades in response to various stressors, especially stressors that are closely related to stomatal movements, such as drought stress.

Although it has been shown that *SpMPK1*, *SpMPK2*, and *SpMPK3* positively regulate the responses of tomato plants to many biotic stresses, such as insects and bacteria, their functions under abiotic stresses are poorly understood. Here, we studied the functions of *SpMPK1*, *SpMPK2*, and *SpMPK3* in the drought tolerance of wild-type *Solanum pimpinellifolium* plants using the virus-induced gene silencing (VIGS) method. The loss-of-function studies indicate that *SpMPK1*, *SpMPK2*, and *SpMPK3* may play positive roles in drought stress tolerance in tomato via controlling ABA-induced stomatal movements and H_2_O_2_ production.

## 2. Results

### 2.1. Silencing *SpMPK1*, *SpMPK2*, and *SpMPK3* Reduced the Drought Tolerance of Tomato Plants

**Figure 1:**
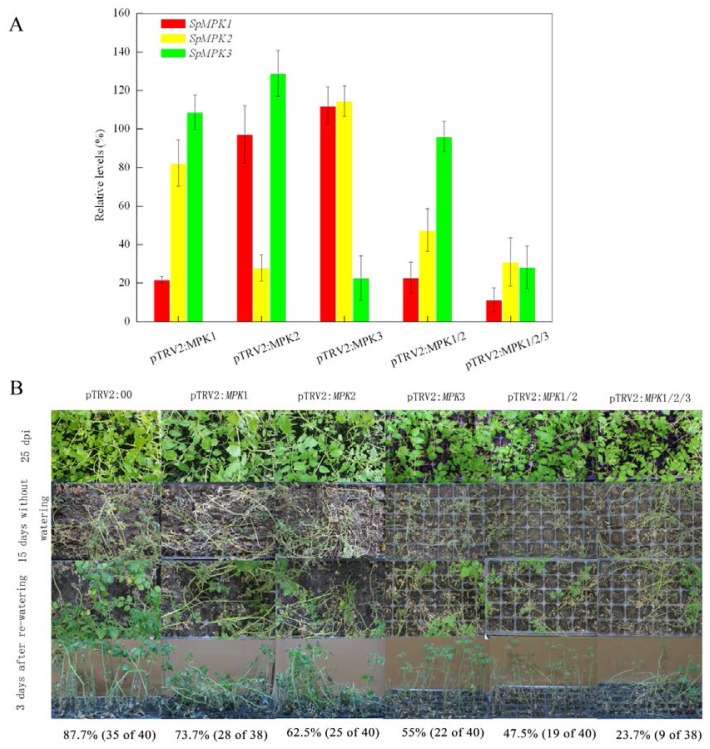

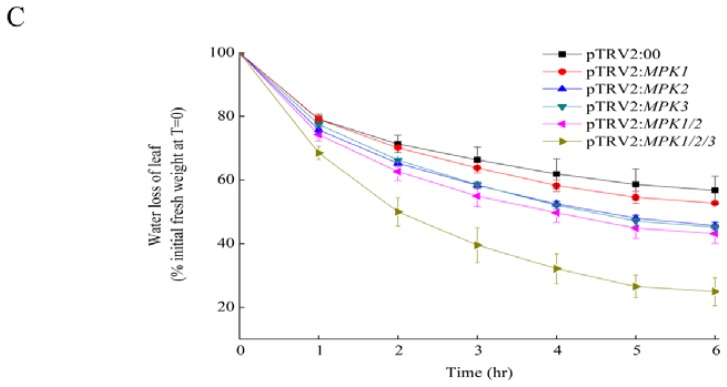
Silencing *SpMPK1*, *SpMPK2*, and *SpMPK3* attenuated the drought tolerance of tomato plants. (**A**) Transcription levels of *SpMPK1*, *SpMPK2*, and *SpMPK3* in each type of gene-silenced plants. Nine to 10-day-old plants were infiltrated with *A. tumefaciens* containing pTRV2:*MPK1*, pTRV2:*MPK2*, pTRV2:*MPK3*, pTRV2:*MPK1/2*, pTRV2:*MPK1/2/3* and pTRV2:00 (control). *SpMPKs* gene silencing efficiency was analyzed for mRNA levels by quantitative RT-PCR at 25 dpi. The transcription levels in VIGS plants (*n* ≥ 50) were expressed as percentages of the mean levels in control plants, which were defined as 100%; (**B**) Drought sensitivity of gene-silenced plants. Gene-silenced plants at 25 to 30 dpi were further grown for 15 day without watering and consequently re-watered for 3 day, and then the surviving plants were counted; (**C**) Measurement of water loss of detached leaves. Detached leaves of gene-silenced plants were weighed at the indicated times after their excision. Water loss was calculated as the percentage of initial fresh weight. The data represent means ± SD of 10 leaves from each of three replicates.

Gene-silenced plants were generated with VIGS constructs. VIGS efficiency was assessed using quantitative reverse transcriptase polymerase chain reaction (qRT-PCR) by analyzing the transcription levels of *SpMPK*1, *SpMPK2*, and *SpMPK3* in the gene-silenced plants compared with those in the control plants. After silencing single genes, the transcription levels of the *MPK* gene targets were reduced by 80% (*SpMPK1*), 73% (*SpMPK2*), and 78% (*SpMPK3*), but no reduction was observed in the transcription levels of other *MPKs* (Figure 1). For the co-silencing assays, the transcription levels of *SpMPK1* and *SpMPK2* in plants having both *SpMPK1/SpMPK2* genes silenced were reduced by 77% and 65%, respectively. For plants with all three genes co-silenced, the transcription levels of *SpMPK1*, *SpMPK2*, and *SpMPK3*, were reduced by 89%, 70%, and 70%, respectively. These results suggest that endogenous *SpMPK*1, *SpMPK2*, and *SpMPK3* genes were successfully silenced in the experimental plants.

To examine the drought tolerance of plants that underwent individual gene silencing (*SpMPK1*, *SpMPK2*, or SpMPK3) and combined gene silencing (*SpMPK1*/*SpMPK2* or *SpMPK1/SpMPK2*/*SpMPK3*), those containing <50% of the target gene transcripts were grown for an additional 15 days without watering, until most of the gene-silenced plants wilted. These plants were then re-watered for three days and their survival rates were compared. After re-watering, the individually gene-silenced plants showed lower survival rates—73.7% (28 of 38), 62.5% (25 of 40), and 55% (22 of 40) for plants with the silenced *SpMPK1*, *SpMPK2*, and *SpMPK3* genes—respectively-compared with the control plants, whose survival rate was 87.5% (35 of 40). In contrast, plants with co-silenced *SpMPK1*/*SpMPK2* genes had a survival rate of only 47.5% (19 of 40), while those with co-silenced *SpMPK1/SpMPK2*/*SpMPK3* genes had a survival rate of only 23.7% (9 of 38) after re-watering.

To further evaluate the responses of gene-silenced plants to drought stress, we examined water losses in detached leaves. As shown in Figure 1C, leaves from the individually gene-silenced plants lost more water than leaves of the control plants. After a 6-h incubation, control leaves lost 43% ± 4.4% of their initial weight, whereas leaves from the individually silenced *SpMPK1*, *SpMPK2*, and *SpMPK3* plants lost 47% ± 0.5%, 54% ± 1.2%, and 55% ± 1.5%, respectively, of their initial weights. A similar outcome was seen for the leaves of *SpMPK1*/*SpMPK2* co-silenced plants, which lost 57% ± 3.0% of their initial weight. Leaves from the *SpMPK1/SpMPK2*/*SpMPK3* co-silenced plants lost water faster than the others, losing 31% ± 2.1% of their initial weight in the first hour after being detached; after 6 h of incubation, the leaves lost approximately 75% ± 4.5% of their initial weight, which was a significantly greater loss than was seen in the other experimental plants (*p* < 0.02). As a whole, detached leaves from gene-silenced plants lost more water than those of the control plants after 6 h of incubation.

### 2.2. Silencing SpMPK1, SpMPK2, and SpMPK3 Impaired Stomatal Closure in Response to Abscisic Acid (ABA) and Hydrogen Peroxide (H_2_O_2_)

**Figure 2:**
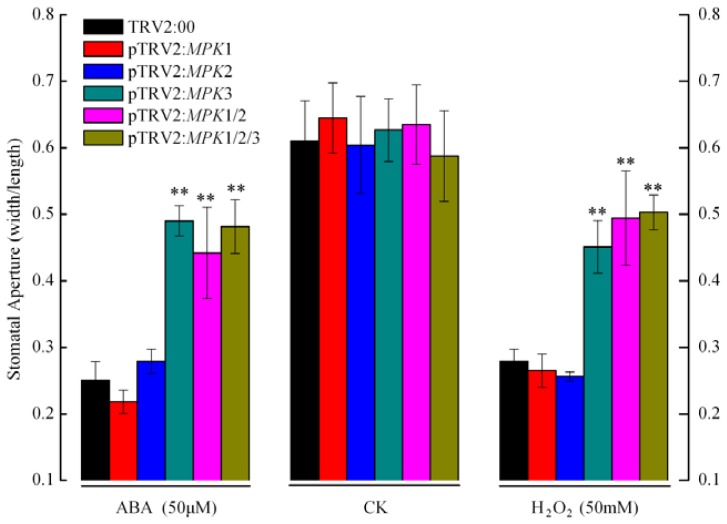
Stomatal apertures (the ratio of width to length) of gene-silenced plants in response to ABA and H_2_O_2_. Leaves of control, individual gene-silenced (*SpMPK1*, *SpMPK2*, or *SpMPK3*) and combined gene-silenced (*SpMPK1*/*SpMPK2* or *SpMPK1/SpMPK2*/*SpMPK3*) plants were incubated in stomatal opening solution for 2 h. Some of them were transferred to solutions containing 50 μM ABA or 50 mM H_2_O_2_ for 2 h and the others remained in the opening solution for control (CK). Stomata on the adaxial surface were observed by light microscopy. For each type of gene-silenced plants, at least 20 stomata from each sample of ten genes-silenced plants were measured. The data represent means ± SD. Asterisks indicate that the mean value is signficantly different from the control at *p* < 0.0001.

Stomata are pores formed by pairs of guard cells that are located in the epidermis. Plants can tolerate drought stress by regulating the aperture of the stomata to minimize water losses caused by transpiration. ABA-deficient plants or those with impaired ABA signaling are unable to adaptively regulate their stomatal apertures and are highly susceptible to drought stress [13,27,28]. To investigate whether *SpMPK1*, *SpMPK2*, and *SpMPK3* genes function in ABA- and H_2_O_2_-sensitive stomatal responses, we examined the ABA-dependent stomatal activity in our gene-silenced plant models. Leaves from the control and experimental plants were submerged in stomatal opening solution and then treated with 50 μM ABA or 50 mM H_2_O_2_ for 2 h, and then stomatal apertures were measured. In the stomatal opening solution, all of the guard cells were fully opened prior to ABA and H_2_O_2_ exposure (Figure 2, CK). However, in the presence of ABA and H_2_O_2_, the control plants and the individually silenced *SpMPK1* and *SpMPK2* plants were highly sensitive to ABA and H_2_O_2_, which caused their stomata to close, as shown in Figure 2. In contrast, stomatal closure was markedly impaired in the leaves of *SpMPK1*/*SpMPK2* co-silenced plants, as compared with that of the individual *SpMPK1* and *SPMPK2* silenced plants (*p* < 0.0001). These results suggest that *SpMPK1* and *SpMPK2* may redundantly regulate ABA- and H_2_O_2_-induced stomatal closure. In addition, individual *SpMPK3* gene-silenced plants and *SpMPK1*/*SpMPK2*/*SpMPK3* co-silenced plants exhibited the same stomatal behavior as *SpMPK1*/*SpMPK2* co-silenced plants when exposed to ABA and H_2_O_2_. These results indicate that the function of *SpMPK1* and *SpMPK2* may overlap with that of *SpMPK3* in regulating ABA- and H_2_O_2_-induced stomatal closure.

### 2.3. SpMPK3 is Involved in Abscisic Acid (ABA)-Induced Hydrogen Peroxide (H_2_O_2_) Production by Regulating the Expression of *CAT1*

**Figure 3:**
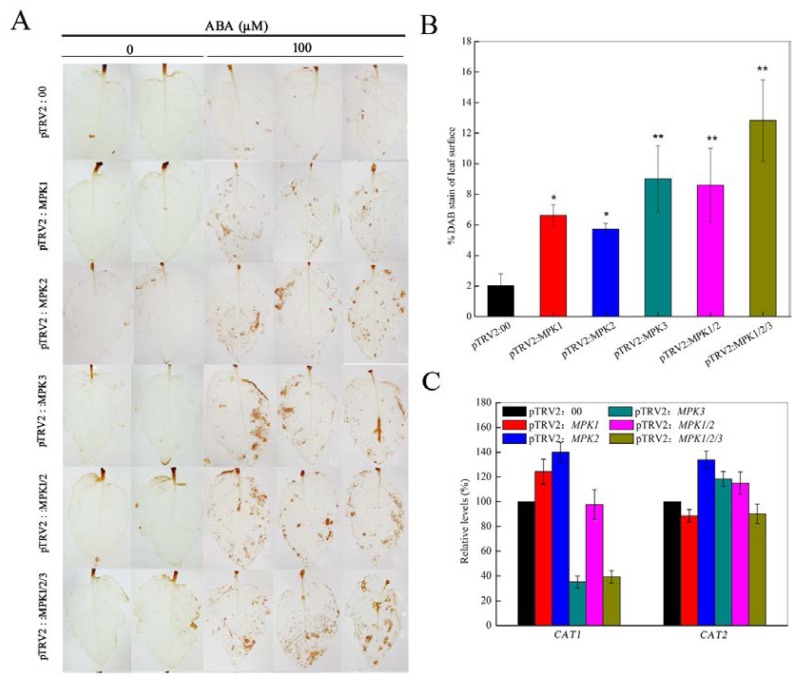
H_2_O_2_ production and *CATs* transcription levels of gene-silenced plants. (**A**) Detection of ABA-induced H_2_O_2_ production by DAB staining. Leaves of control, individual gene-silenced (*SpMPK1*, *SpMPK2*, or *SpMPK3*) and combined gene-silenced (*SpMPK1*/*SpMPK2* or *SpMPK1/SpMPK2*/*SpMPK3*) plants were treated with 100 μM ABA for 2 h and transferred to 100 mg·mL^−1^ DAB solution for 8 h. The presence of H_2_O_2_ in the leaves was visualized as a dark brown color; (**B**) The amount of H_2_O_2_ in leaves of gene-silenced plants. The data represent means ± SD of two leaves from each of six gene-silenced plants, respectively. Asterisk, *p* < 0.05; double asterisks, *p* < 0.01; (**C**) The transcription levels of H_2_O_2_ scavenging genes (*CAT1* and *CAT2*) in VIGS plants treated with 100 μM ABA. The experiments are representative of 10 gene-silenced plants, respectively.

According to previous studies, ROS is also involved in the ABA signal transduction pathway that regulates guard cells [29,30]. The above results prompted us to wonder whether or not *SpMPKs* act on ABA-ROS signaling. Therefore we used our gene-silenced plant models to investigate how *SpMPK1*, *SpMPK2*, and *SpMPK3* influence ABA-induced H_2_O_2_ production. To do this, the H_2_O_2_ levels in the gene-silenced plants were measured when plants were exposed to ABA. As shown in Figure 3A, only basal levels of H_2_O_2_ were detected in non-treated control plants, but H_2_O_2_ production increased substantially in response to ABA exposure. In the presence of 100 μM ABA, H_2_O_2_ production in individually silenced *SpMPK1* and *SpMPK2* plants increased by 6.6% and 5.7%, respectively, which was significantly more than the increase seen in the control plants (*p* < 0.05) (shown in Figure 3B). The individually silenced *SpMPK3* plants and both groups (*SpMPK1/SpMPK2* and *SpMPK1/SpMPK2/SpMPK3*) of co-silenced plants displayed a hypersensitivity to ABA, evidenced by increases of 9%, 8.5% and 12.8%, respectively, in H_2_O_2_ production, which were significantly greater than those of plants with individually silenced *SpMPK1* and *SpMPK2* genes (*p* < 0.01). The data presented here demonstrate that *SpMPK1*, *SpMPK2*, and *SpMPK3* may be involved in ABA-H_2_O_2_ signaling related to stress tolerance by regulating the production of H_2_O_2_. Future studies are needed, because it is not yet known whether H_2_O_2_ acts upstream or downstream of the *SpMPKs*.

Catalase, produced by the expression of the *CAT* genes, is an important enzyme that catalyzes the decomposition of H_2_O_2_. However, in the ABA signaling pathway, the transcription levels of *CAT1* were reduced in individually silenced *SpMPK3* plants and in *SpMPK1/SpMPK2/SpMPK3* co-silenced plants, but not in the other gene-silenced plants. Furthermore, we did not observe a significant decrease of *CAT2* transcription levels in any of the gene-silenced plants (Figure 3C). The lack of correlation between *CAT* transcriptional levels and the H_2_O_2_ levels of gene-silenced plants suggests that ABA-mediated H_2_O_2_ production may be governed by multiple genes that function in different pathways. These results suggest that *CAT1* may be involved in the feedback regulation of H_2_O_2_ signaling and that *SpMPK3* may regulate the H_2_O_2_ signaling by mediating the expression of *CAT1*.

## 3. Discussion

Drought is one of the greatest threats to agriculture production worldwide. Previous findings have shown that MAPKs are involved in the tolerance-related signaling networks associated with various stressors, including drought stress. In this study, we found that inhibiting the function of *SpMPK1*, *SpMPK2*, and *SpMPK3* genes in tomato plants reduced their tolerance to drought.

As is commonly known, the movement of stomata is tightly linked to water loss. In drought conditions, plants produce ABA, which then triggers a signaling cascade that causes stomata to close, thus preventing water loss [31]. Therefore, stomatal behavior may reflect a plant’s tolerance for drought stress. In this study, the functions of *SpMPK1*, *SpMPK2*, and *SpMPK3* as they relate to stomatal behavior were investigated. Although inhibiting *SpMPK1*, *SpMPK2*, and *SpMPK3* resulted in the formation of clustered stomata, we did not observe remarkable differences between the control plants and the gene-silenced plants (both the individually silenced and co-silenced plants) (shown in Figure S1). These results suggest that additional *MPKs* besides *SpMPK1*, *SpMPK2*, and *SpMPK3*, may exist in the tomato that redundantly regulates stomatal development. However, all the *SpMPKs* investigated here participated in ABA- and H_2_O_2_-induced stomatal closure, and the function of *SpMPK1* may be redundant with that of *SpMPK2*. It has been reported that a given MAPKKK or MAPKK can activate more than one target kinase, which means that MAPKKKs and MAPKKs function as divergence points in signal transduction [32]. Thus, plants can produce thousands of different combinations of MAPK cascades to regulate stress responses, such that many of the cascades overlap [33]. In tomato plants, MAPKs are generally grouped into four subfamilies [34]. *SpMPK1*, *SpMPK2*, and *SpMPK3* belong to the same subfamily (subfamily A) because their sequences are highly homologous. Therefore, functional redundancies between *SpMPK1*, *SpMPK2*, and *SpMPK3* are unsurprising.

Previous studies have shown that ABA can activate *AtMPK3* and *AtMPK6* in *Arabidopsis* species [15–17,35]. In agreement with these results, we found that *SpMPK3* and *SpMPK1/2* (homologous to *AtMPK3* and *AtMPK6*) were also up-regulated in the presence of ABA (Figure S2). Additionally, silencing *SpMPK1*, *SpMPK2*, and *SpMPK3* impaired ABA-induced stomatal closure. Thus, it is speculated that these genes act downstream of ABA by regulating ABA signaling to mediate drought tolerance in tomato plants. In addition, H_2_O_2_ is known to be an intracellular messenger that can evoke specific cellular responses under different stimuli [36–39]. Previous studies have shown that ABA triggers the production of H_2_O_2_, and H_2_O_2_ mediates ABA-induced stomatal closure; these processes are thought to be regulated by MAPK signaling [26,40]. In this study, we demonstrated that silencing *SpMPK1*, *SpMPK2*, and *SpMPK3* worked to impair stomatal closure, indicating that *SpMPK* genes may act downstream of H_2_O_2_. However, we also observed increased H_2_O_2_ levels in gene-silenced plants exposed to ABA, which alternatively suggests that *SpMPK1*, *SpMPK2*, and *SpMPK3* may be involved in ABA-induced H_2_O_2_ production. In addition, silencing the *SpMPK3* gene attenuated the transcription level of *CAT1*, the translated product of which scavenges H_2_O_2_. These results demonstrate that *SpMPK3* is involved in the feedback regulation of H_2_O_2_ production and *CAT1* expression. For *SpMPK1* and *SpMPK2*, increased levels of H_2_O_2_ were observed in the individually silenced and co-silenced plants, but *CAT* transcription levels were not affected, which suggests that some unknown H_2_O_2_-scavening genes may exist in *SpMPK1/SpMPK2*-mediated H_2_O_2_ signaling.

MAPK cascades represent the primary way in which plant cellular functions are controlled in response to a number of external signals. As shown in our results, the complementary and commutable functions of *SpMPK1*, *SpMPK2*, and *SpMPK3* in drought tolerance reveal the complexity and crosstalk associated with MAPK cascades. Because MAPKs are regulated post-translationally by phosphorylation and they act on various substrates, the loss of a functional gene product might not pinpoint the function of a MAPK cascade. Therefore, a complementary gain-of-function assay on *SpMPK*s would help us understand the complex roles of *SpMPK*s in the tolerance responses of tomatoes. Although 16 MAPK family genes have been identified in tomatoes to date, no systematic investigations on MAPKK and MAPKKK family genes have yet been performed. Thus, identifying different combinations of MAPKKK-MAPKK-MAPK cascades under stimulus-specific stressors would be a highly complex undertaking.

## 4. Experimental Section

### 4.1. Plant Material and Growth Conditions

Seeds of *S. pimpinellifolium* L03708, kindly supplied by AVRDC—The World Vegetable Center, were grown in cell trays filled with matrix in artificial climate incubator at 21 ± 2 °C with 50% relative humidity and a 16 h photoperiod with light intensity ranging from 300 to 400 μmol·m^−2^·s^−1^. Nine- to 10-day-old seedlings (cotyledons fully expanding and true leaves just emerging) were subjected to VIGS tests by inoculation with *Agrobacterium* suspensions (as shown below).

### 4.2. VIGS Constructs

The pTRV1 and pTRV2 VIGS vectors described in Liu *et al*. [41] were obtained from Dr. Dinesh-Kumar (Yale University, New Haven, CT, USA). Fragments for individually and combined silencing *SpMPK1*, *SpMPK2*, *SpMPK3* were obtained by polymerase chain reaction (PCR) using cDNA synthesized with PrimeScript RT reagent Kit (Takara, Dalian, China) from total RNA extracted from leaf tissues of L03708 using Trizol reagent (Invitrogen, Carlsbad, CA, USA) as a template and the primer pairs listed in Table S1. The PCR products were cloned into the pMD-18T vector, and then recombined into pTRV2 plasmid digested by *Xho* I/*Sac* I. The recombinant constructs were electroporated into cells of *Agrobacterium tumefaciens* GV3101. The resultant *Agrobacterium* strains were used in the indicated VIGS experiments.

### 4.3. Infiltration of pTRV-Containing *A. tumefaciens* Cultures into Cotyledons

*A. tumefaciens* GV3101 strains carrying each TRV derivative were grown at 28 °C in LB medium containing antibiotics (50 μg/mL of kanamycin, 10 μg/mL of rifampicin and 50 μg/mL of gentamycin), and bacterial suspensions (OD_600_ = 2) were prepared for infiltration on cotyledons following a similar procedure as described in Velásquez *et al*. [42]. The cotyledons of young seedlings were infiltrated with *Agrobacteria* by using a 1-mL syringe. Agroinfiltrated plants were maintained at 18–22 °C in a growth chamber with a 16 h light period for the indicated periods of time.

### 4.4. RNA Isolation and Quantitative RT-PCR

Total RNA was isolated using Trizol reagent (Invitrogen, Carlsbad, CA, USA) and then treated with DNase I (Fermentas, Glen Burnie, MD, USA) to clean out DNA. cDNA synthesized from 1 μg of total RNA using the PrimeScript RT reagent Kit (Takara, Dalian, China) was utilized for quantitative RT-PCR. Quantitative RT-PCR was performed using SYBR Premix Ex Taq II (TaKaRa, Dalian, China) on iQ5 Real-Time PCR Detection System (BIO-RAD Corp., Hercules, CA, USA). The PCR cycling conditions were as follows: 95 °C for 1 min, followed by 40 cycles of 95 °C for 10 s, 55 °C for 10 s and 72 °C for 20 s. The melting curve was routinely performed after 40 cycles to verify primer specificity. The 2^−ΔΔCt^ method was applied to calculate the change of expression of each gene [43]. *S. pimpinellifolium* elongation factor 1-α (*EF*1α) mRNA level was used as internal control for normalization [44]. All primer sequences are given in Table S1.

### 4.5. Measurement of Stomatal Aperture

Leaves of control, individual gene-silenced (*SpMPK1*, *SpMPK2*, or *SpMPK3*) and combined gene-silenced (*SpMPK1*/*SpMPK2* or *SpMPK1/SpMPK2*/*SpMPK3*) plants at 25–30 dpi were used for the observation of matured stomata. Detached leaves were incubated in stomata opening solution (10 mM MES, pH 6.1, in a 50 mM KCl solution), and illuminated with 250 to 350 μmol m^−2^ s^−1^ light for 2 h and then transferred to the stomatal opening solution supplemented with 50 μM ABA or 50 mM H_2_O_2_ respectively [45]. Subsequently, epidermal strips were carefully prepared from the adaxial surface of the leaf, mounted on glass slides and observed using an Olympus PD72 microscope (Olympus Corp., Tokyo, Japan). The ratio of width to length of the stomata was measured using Image Pro Plus software (Media Cybernetics Inc., Rockville, MD, USA). Over 20 guard cells from each sample of ten gene-silenced plants were used to measure stomatal aperture.

### 4.6. DAB Staining

DAB staining was used to observe the accumulation of H_2_O_2_[32,46]. Leaves of individual and combined gene-silenced plants at 25–30 dpi were treated with 100 μM ABA for 2 h, then incubated with 1 mg·mL^−1^ DAB solution for 8 h. Following the incubation, DAB-stained samples were submerged in bleaching solution (ethanol:acetic acid:glycerol, 3:1:1) and boiled in a water bath for 15–20 min to remove chlorophyll. The presence of H_2_O_2_ in leaves was visualized as a dark brown color.

### 4.7. Drought Tolerance Assay

Gene-silenced plants at 25–30 dpi were subjected to drought stress by ceasing watering for 15 days. After re-watering for the following three days, surviving plants were counted. For the experiment of water loss, detached leaves were placed in petri dishes at room temperature with approximately 60% humidity under dim light. The weights of leaves were measured over time (0–6 h). Water loss of leaf was expressed as a percentage of initial fresh weight.

### 4.8. Tissue Sampling for Assays

Leaf samples for RNA analysis, water loss of leaves, and stomatal aperture or DAB staining assays were collected at 25–30 dpi. The development and extent of gene silencing were assessed by monitoring the photo-bleaching pattern of PHYTOENE DESATURASE-silenced plants according to Liu *et al*. [41]. For each plant, the fifth to ninth true leaves were used for their high silencing efficiency (shown in Figure S3). Samples for RNA analysis, five leaflets from the true leaves (fifth-ninth), were collected and frozen immediately, whereas the other leaflets (total of 15–25) were utilized for the other assays.

### 4.9. Statistical Analysis

R 2.15.2, an open-source software, is used for the statistical analysis. The data were subjected to univariate ANOVA analysis of variance followed by a *post hoc* test. Values were computed as the means ± SD of three or more independent experiments.

## 5. Conclusions

In conclusion, plants with individually silenced *SpMPK1*, *SpMPK2*, and *SpMPK3* genes, as well as co-silenced *SpMPK1/SpMPK2* and *SpMPK1/SpMPK2/SpMPK3* genes, had lower survival rates than control plants when they were exposed to drought-like conditions. In addition, silencing these *SpMPKs* resulted in impaired stomatal closure and increased H_2_O_2_ production when plants were exposed to ABA. In conclusion, this study demonstrates that *SpMPK1*, *SpMPK2*, and *SpMPK3* may play important roles in the drought tolerance of tomato plants. In drought tolerance, the functions of *SpMPK1* and *SpMPK2* may be redundant, and they may overlap with that of *SpMPK3*. Future studies will employ genetic transformation experiments to further explore how *SpMPKs* function in the drought tolerance of tomato plants.

## Supplementary Information
