# Supplementary material for: Silencing the SpMPK1, SpMPK2, and SpMPK3 Genes in Tomato Reduces Abscisic Acid—Mediated Drought Tolerance
Source: Int J Mol Sci. 2013 Nov 6;14(11):21983–96. doi: 10.3390/ijms141121983 (PMC3856046; doi:10.3390/ijms141121983)
Supplement: Supplementary file 1 [file ijms-14-21983-s001.pdf]

Supplementary Information

Table S1. Primers used in this study.

Primer code	Primer sequences (5'–3')	GenBank accession no.	Products lengths (bp)	Objectives
COSM121F	GGCGCGAGCTCCATGGTGGCAGGTTCATTC	AY261512	577	For cosilencing of *SpMPK1/2*
COSM121R	CGGCGCTCGAGGCTCAGGTGGACGATACCAT			
SM1F	GGCGCGAGCTCATAATTGCTGACAGATTGTTGC	AY261512	200	To specifically silence *SpMPK1*
SM1R	CGGCGCTCGAGCATTTCAGTCTAAAATAAAATCCAC			
SM2F	GGCGCGAGCTCGTACTCGCTCGTTTGCTGTT	AY261513	214	To specifically silence *SpMPK2*
SM2R	CGGCGCTCGAGCATTTCTGGAACTAAAAATACAGAT			
SM3F	GGCGCGAGCTCGCATAAGAGAAATCAGTTCTTCTCT	AK319930	409	To specifically silence *SpMPK3*
SM3R	CGGCGCTCGAGACACCCAAAACTTCAAAATGAC			
COSM1231F	GGCGCGAGCTCACTGATCTCCATCAAATTATTCG	AY261512	252	For cosilencing of *SpMPK1/2/3*
COSM1231R	GCTCTAGAAGGTGGACGATACCATCTTG			
COSM1233F	AATTGTCACTCATGCTGGACA	AY261514	220	
COSM1233R	CGGCGCTCGAGAATTTCACGGAGCGTCCT			
SPDSF	GGCGCGAGCTCGGCACTCAACTTTATAAACC	NM_001247166	409	To silence *SpPDS*
SPDSR	CGGCGCTCGAGCTTCAGTTTTCTGTCAAACC			
EF1αF	GACAGGCGTTCAGGTAAGG	X14449	127	To assess the transcription levels of *SpEF1α*
EF1αR	CCAATGGAGGGTATTCAGC			
qM1F	ATCCCAGAAGGAGAATAACAG	AY261512	228	To assess the transcription levels of *SpMPK1*
qM1R	ATCAAACCTGcAACAATCTG			
qM2F	ATTCCACCACCTCAACGA	AY261513	196	To assess the transcription levels of *SpMPK2*
qM2R	TGCTAGGCTTCAAGTCCC			
qM3F	GCAACTCCCACAACATCC	AY261514	233	To assess the transcription levels of *SpMPK3*
qM3R	TCTGCTCTTCTCCTATCCCT			
qPDSF	ATGCCACGACCAGAAGAT	NM_001247166	200	To assess the transcription levels of *SpPDS*
qPDSR	TGCTGTAGACAAACCACCC			
qCAT1F	GTGGATTATTTGCCCTCG	NM_001247898	152	To assess the transcription levels of *SpCAT1*
qCAT1R	GGTTCCCATGATCTGTACCTC			
qCAT2F	TCTGAAGCCAAATCCTAAGTC	NM_001247257	109	To assess the transcription levels of *SpCAT2*
qCAT2R	CAATATCGTCGAAGAGGAAAGT			

Note: words in red are restriction sites added to Forward primers and Reverse primers.

Figure S1. Stomatal Development in control (pTRV2:00) and individual and combined gene-silenced plants. (**A**) The distribution of stomata on the leaves of experimental plants. Control plants follow the one-cell spacing rule (**a**); For gene-silenced plants, clustered stomata are formatted in leaves' lower epidermis (**b–f**); The order is pTRV2:*MPK1*, pTRV2:*MPK2*, pTRV2:*MPK3*, pTRV2:*MPK1/MPK2*, pTRV2:*MPK1/MPK2/MPK3*). Bar = 20 μm in (**a–f**); and (**B**) Stomatal index of the adaxial surface of experimental leaves. Stomatal index (SI) is calculated using the formula proposed by Salisbury (1927): $SI = [S/(E + S)] \times 100\%$, where S equals the number of stomata per unit of leaf area and E equals the number of epidermal cells in the same unit area. At least 3 visions from each sample of ten gene-silenced plants were measured. Data represent means ± SD. Unexpectedly, there is no significant difference between the 5 types of gene-silenced plants and control plants.

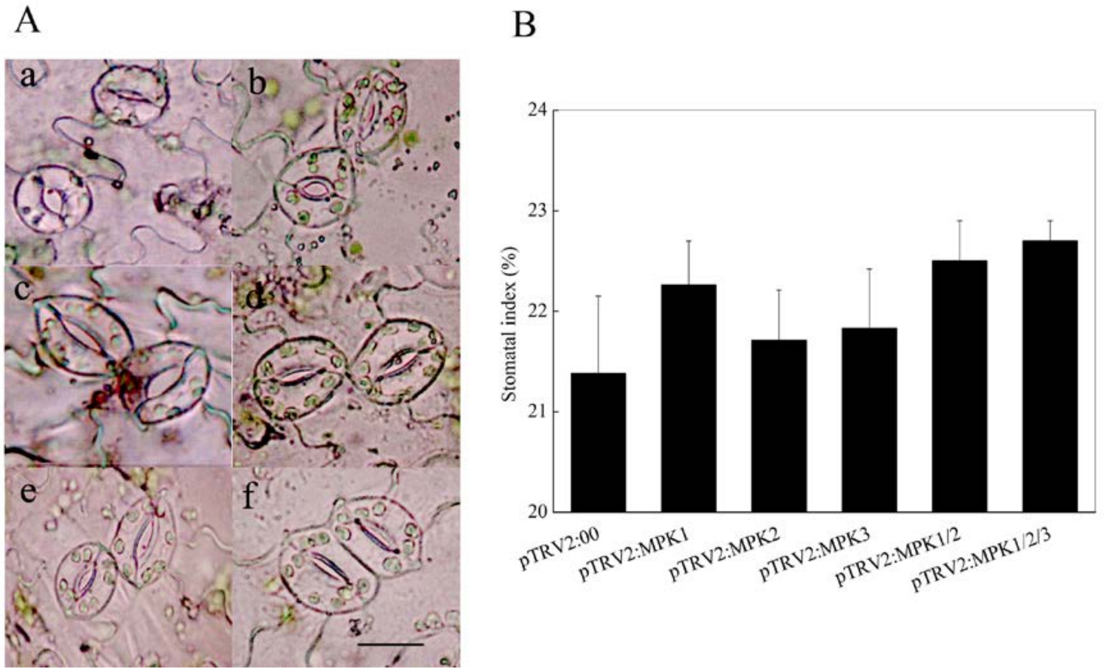

Figure S2. Real-time qRT-PCR analysis of ABA induction of *SpMPK1*, *SpMPK2* and *SpMPK3* genes. 40-day-old wild-type plants were treated with 150 μM ABA for 6 h. Total RNA was obtained from treated plants and analyzed by qRT-PCR using the gene-specific primers listed in Table S1. The graphs indicate the induction fold of the *SpMPK1*, *SpMPK2* and *SpMPK3* genes in response to ABA (150 μM) as compared with that of control (0 μM ABA). The mean value of three technical replicates was normalized to the levels of elongation factor 1-α subunit mRNA, an internal control.

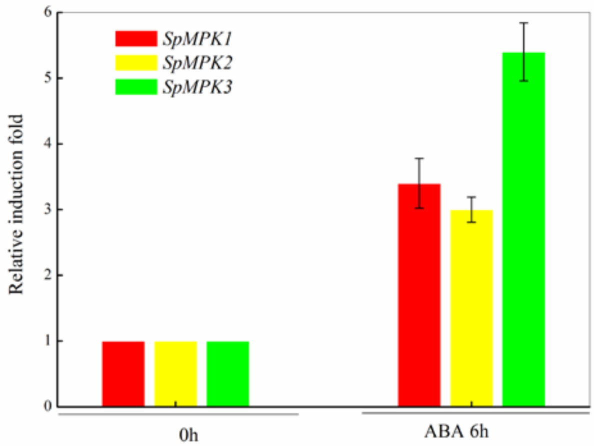

Figure S3. Silencing efficiency of *SpPDS* in wild-type tomato plants. (**A**) The photobleaching phenotype induced by pTRV:*PDS* in *Solanum pimpinellifolium*; (**B**) Leaf samples for RNA analysis come from the fifth to the ninth true leaves; and (**C**) Qualification of silencing efficiency. Leaf silencing efficiency is calculated by comparing the number of a certain true leaf that showed symptoms with the total number of plants inoculated with pTRV2:*PDS* 40 days post-inoculation. The experiments were representative of all the silenced plants ($n \geq 50$ at $OD_{600} = 2.0$). Quantitative RT-PCR was performed to evaluate the gene silencing efficiency and it was presented by the reduction of *SpPDS* transcription levels in VIGS plants compared to control plants which were defined as 100%.

A

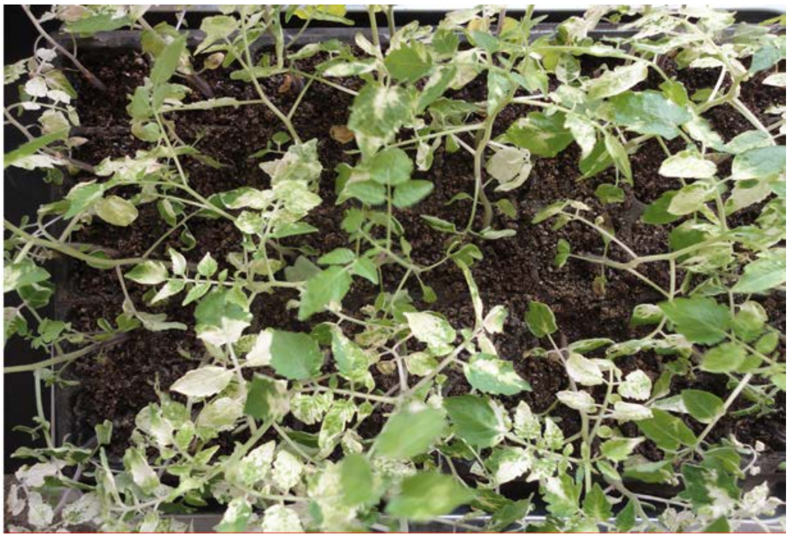

Figure S3. *Cont.*

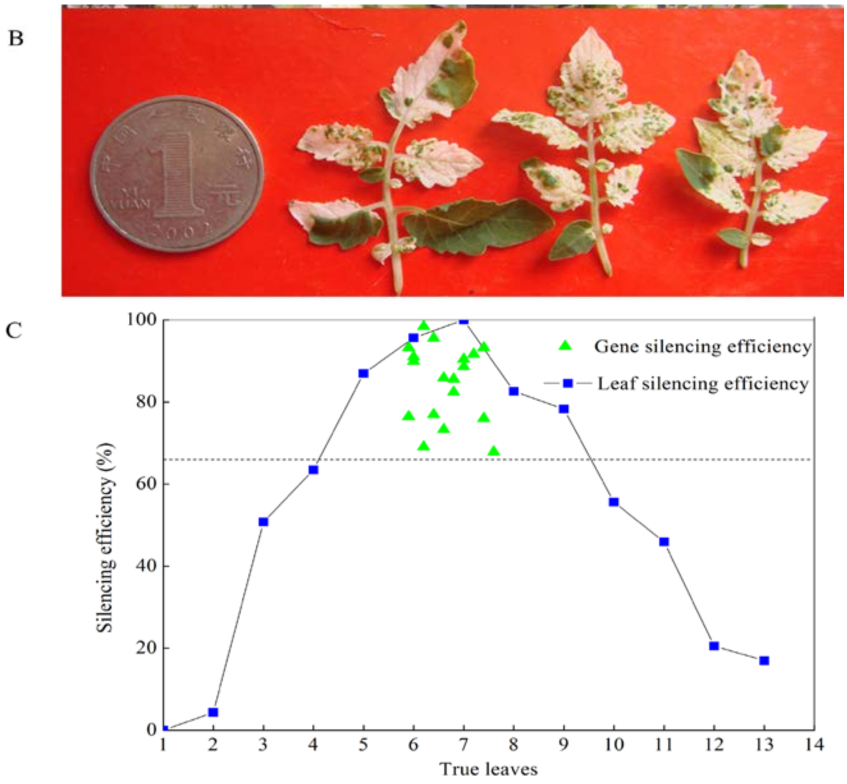

© 2013 by the authors; licensee MDPI, Basel, Switzerland. This article is an open access article distributed under the terms and conditions of the Creative Commons Attribution license (http://creativecommons.org/licenses/by/3.0/).
